# Supplementary material for: A “grappling hook” interaction connects self-assembly and chaperone activity of Nucleophosmin 1
Source: PNAS Nexus. 2023 Jan 6;2(2):pgac303. doi: 10.1093/pnasnexus/pgac303 (PMC9896144; doi:10.1093/pnasnexus/pgac303)
Supplement: pgac303_Supplemental_File [file pgac303_supplemental_file.pdf]

Supplementary Information to:

A “grappling hook” interaction connects self-assembly and chaperone-like activity of Nucleophosmin 1

Mihkel Saluri, Axel Leppert, Genis Valentin Gese, Cagla Sahin, Dilraj Lama, Margit Kaldmäe, Gefei Chen, Arne Elofsson, Timothy M. Allison, Marie Arsenian-Henriksson, Jan Johansson, David P. Lane, B. Martin Hällberg, and Michael Landreh

This SI file contains:
Materials and Methods
Table S1
Figures S1-S8

Materials and Methods

Protein sequences

Hexahistidine (H10) affinity tag: MGHHHHHHHHHH

Tobacco etch virus (TEV) cleavage (/) site: ENLYFQ/S

$NPM1_{FL}$

UniProtKB - P06748 (NPM_HUMAN) Acidic tracts 1, 2, and 3 are shown in bold. Positions 120, 188, and 240 for $NPM1_{120}$, $NPM1_{188}$, and $NPM1_{240}$ are indicated.

^{1}MEDSMDMDMSPLRPQNYLFGCELKADKDYHFKV**DNDENE**HQLSLRTVSLGAGAKDE
LHIVEAEAMNYEGSPIKVTLATLKMSVQPTVSLGGFEITPPVVLRLKCGSGPVHISGQHL
VAV**E**^{120}**EDAESEDEEEED**VKLLSISGKRSAPGGGSKVPQKKVKLAA**DEDDDDDDEEDD**
DEDDDDDDFDDEEAEE^{188}KAPVKKSIRDTPAKNAQKSNQNGKDSKPSSTPRSKGQES
FKKQEKTPKTPKG^{240}PSSVEDIKAKMQASIEKGGSLPKVEAKFINYVKNCFRMTDQEAIQ
DLWQWRKSL^{294}

$NPM1_{AML}$

Mutated residues are underlined.

^{1}MEDSMDMDMSPLRPQNYLFGCELKADKDYHFKVDNDENEHQLSLRTVSLGAGAKDE
LHIVEAEAMNYEGSPIKVTLATLKMSVQPTVSLGGFEITPPVVLRLKCGSGPVHISGQHL
VAVEEDAESEDEEEEDVKLLSISGKRSAPGGGSKVPQKKVKLAADEDDDDDDEEDDD
EDDDDDDFDDEEAEEKAPVKKSIRDTPAKNAQKSNQNGKDSKPSSTPRSKGQESFKK
QEKTPKTPKGPSSVEDIKAKMQASIEKGGSLPKVEAKFINYVKNCFRMTDQEAIQDL<u>CL</u>
<u>AVEEVSLRK</u>^{298}

NPM1$_{\Delta IDR}$

MEDSMDMDMSPLRPQNYLFGCELKADKDYHFKVDNDENEHQLSLRTVSLGAGAKDEL
HIVEAEAMNYEGSPIKVTLATLKMSVQPTVSLGGFEITPPVVLRLKCGSGPVHISGQHLV
AVE_PSSVEDIKAKMQASIEKGGSLPKVEAKFINYVKNCFRMTDQEAIQDLWQWRKSL

Protein expression and purification

The genes for MGH10-TEV-NPM1 and its corresponding mutants in the pET26b(+) expression vector were transformed into chemically competent *E. coli* BL21 (DE3) cells. Over-night cultures were inoculated 1:100 to Luria-Bertani medium containing 50 μg/mL kanamycin. The cultures were grown at +37°C to an OD600 of 0.6-0.9 before induction with 0.5 mM isopropyl-β-D-thiogalactopyranoside (IPTG) and overnight expression at +25°C. For each construct, 500-1000 mL of culture was harvested by centrifugation at 6000 × g for 20 min at RT. The pellet was resuspended in 20 mM Tris, pH 8.0 buffer (1 mL for 10 mL of culture) and stored at -20°C overnight. After thawing, the cells were lysed with the addition of 1 mg/mL lysozyme, 10 μg/ml DNAse I and 2 mM $MgCl_2$ and incubated for 1 hour on ice followed by sonication using a probe sonicator (Qsonica, CT) on ice for 5 min, 0.5 s on/1 s off at 30% power. The samples were centrifugated for 15 min at 15 000 × g, 4°C, the supernatant was decanted, and the pellet was resuspended in 20 mM Tris, 500 mM NaCl pH 8 buffer (1 mL per 10 mL culture) sonicated and centrifuged as before. Two further resuspensions with the same parameters were conducted. Samples taken from steps during expression, lysis and purification were analyzed using SDS-PAGE 4-20% Mini-PROTEAN® TGX™ polyacrylamide gels (Bio-rad Laboratories Inc., USA) stained with Coomassie G-250 Brilliant Blue dye. The protein-containing supernatants were supplemented with 20 mM imidazole and 500 mM NaCl if needed, loaded onto 1 mL HiTrap IMAC HP (Cytiva, Sweden) columns charged with Co^{2+} and eluted with a linear gradient of buffer containing 500 mM imidazole, 500 mM NaCl, 20 mM Tris, pH 8. Fractions were analysed using SDS-PAGE gels, protein-containing fractions were pooled, concentrated using appropriate MWCO Amicon ® Ultra 15 centrifugation tubes (Merck, USA) and dialysed at +4°C five times against 20 mM Tris, 500 mM NaCl, pH 8 using Slide-A-Lyzer™ MINI Dialysis Devices with 10K MWCO (Thermo Scientific, USA). Protein concentrations were determined using a Pierce™ BCA assay (Thermo Scientific, USA).

Aβ_{42} aggregation assays

Aβ_{42} was produced as described previously [44]. Aggregation kinetics were monitored in solution by measuring total ThT fluorescence using a POLARstar Omega microplate reader (BMG Labtech, Germany) with an excitation filter at 440 nm and an emission filter at 480 nm. All measurements were conducted at +37°C for 24 hours, without agitation. Samples were prepared with 10 μM ThT in 170 mM NaCl, 17 mM Tris, 3 mM NaPi, 0.03 mM EDTA pH 8 in black half-area 384-well polystyrene microplates with a transparent (Corning, USA). The total reactant volume of each replicate was 20 μl. For kinetic experiments with constant Aβ42 concentrations, a final concentration of 3 μM Aβ42 was

prepared in the absence and presence of varying molar ratios of NPM1 variants. To determine the effects of different NPM1 concentrations on the aggregation half time of Aβ42 fibril formation, fluorescence data was normalized and fitted to an empirical logistic5 function with an equation (1):

$$y = F_0 + \frac{P - F_0}{\left(1 + \left(\frac{x}{x_0}\right)^{-h}\right)^{s}} \tag{1}$$

where F_0 is the baseline value, P the plateau value, x the time point value, x_0 the time factor, h the Hill slope steepness factor, s the control factor. The aggregation half-time τ_0 values were derived from the following equation (2):

$$\tau_0 = \frac{x_0}{10^{\frac{1}{h}\log_{10}\left(2^{\frac{1}{s}}-1\right)}} \tag{2}$$

Where x_0 is the time factor, h the Hill slope steepness factor, s the control factor. Data is represented as the average±standard deviation from 4-5 experiments.

Native mass spectrometry

All purified NPM1 variants were buffer-exchanged into 1 M ammonium acetate, pH 8.0, using Bio-Spin P-6 columns (BioRad, CA). Mass spectra were acquired on a Waters Synapt G1 travelling wave ion mobility mass spectrometer modified for high-mass analysis (MS Vision, NL) equipped with an offline nanospray source. The capillary voltage was 1.5 kV, the source pressure was 8 mbar, and the source temperature was 80°C. Mass spectra were visualized using MassLynx 4.1 (Waters, UK).

AF structure predictions

All AF predictions for NPM1 from *Homo sapiens* were generated using Colab Fold (https://colab.research.google.com/github/sokrypton/ColabFold/blob/main/AlphaFold2.ipynb) Version 1.4 with default settings (5 models, no AMBER step). Inclusion of an AMBER step did not yield noticeably different structures, as did inclusion of the polyhistidine-tag in the NPM1 sequence. Structures were visualized with ChimeraX V1.3 (https://www.cgl.ucsf.edu/chimerax) [45].

Negative stain electron microscopy

For negative stain EM, a final concentration of 3 µM $A\beta_{42}$, 3 µM $NPM1_{240-294}$, or 3 µM $A\beta_{42}$ + 3 µM $NPM1_{240-294}$ were prepared in 160 mM NaCl, 16 mM Tris, 4 mM NaPi, 0.04 mM EDTA pH 8 and incubated overnight at 37°C. The samples were then centrifuged at 17000 x *g* for 30 min and 80% of the supernatant was removed. The remainder was resuspended and 5 µL was loaded to 200 mesh copper grids with Formvar/Carbon support film that had been glow-discharged at 25 mA for 2 min. After one minute of incubation, the liquid was removed and the negative staining was carried out by applying a 5 µl of 1 % (w/v) uranyl acetate (in H_2O) to the grid for 20-30 seconds. Then, the liquid was removed and the

procedure was repeated 6 times [46]. The grids were imaged in Talos 120 C G2 (Thermo Scientific) equipped with a CETA-D detector.

cryo-EM data collection and processing

Four microliters of 0.75 mg/ml of purified NPM1 were applied to Cryomatrix 2/1 grids (glow discharged for 2 min at 25 mA) in a Vitrobot MK IV (Thermo Fisher Scientific) at 4°C and 100% humidity. Sample excess was removed by blotting for 4 s using a blot force of 1 followed by vitrification in liquid ethane.

The data were collected on a Krios G3i electron microscope (Thermo Fisher Scientific) at an operating voltage of 300 kV with Gatan BioQuantum K3 image filter/detector (operated with a 10 eV slit) at the Karolinska Institutet's 3D-EM facility, Stockholm, Sweden. The data were collected using EPU (Thermo Fisher Scientific). An EFTEM SA magnification of 165,000x was used, resulting in a pixel size of 0.505 Å, with a total dose of 54 $e^{-}/Å^{2}$ divided across 60 frames over 2 s (fluency of 6.9 e-/px/sec). Target defocus values were set between -0.2 to -2 μm and using a stage tilt of 0° or -20°. The data processing strategy is schematized in Fig S3a. Motion correction and CTF estimation was performed in Warp [47].The micrographs were then imported in Scipion3 [48] and denoised using Janni for picking with crYOLO [49] using the pre-trained model for de-noised micrographs. The particle picks were pruned using XMIPP Deep Micrograph Cleaner [50,51]. The particles were then extracted in WARP (64 px box, 2-fold binning to 2.02 Å/px). 2D classification of the particle set was performed in CryoSPARC v3.01 [52] and the good 2D classes were manually selected for *ab-initio* reconstruction in CryoSPARC of 1 class. The particles were then refined in RELION 4.0 beta [53].The refined particles were re-extracted in WARP (192 px box, 1.01 Å/px) and further refined in RELION 4.0 beta using a mask (covering N-terminal and C-terminal domains) and subjected to 3D classification without alignment (6 classes, T 20) using the same mask. The class with very weak density for C-terminal domains (Class 1) and the class with the strongest density for C-terminal domains (Class 6) were then selected and refined individually in CryoSPARC v3 non-uniform refinement. In order to improve the resolution on the N-terminal region and be able to fit an atomic model for this region, Class 6 was further subjected to 3D classification in RELION 4.0 beta. The two classes which reconstructed to the highest resolution were pooled and re-extracted in WARP (382 px box, 0.505 Å/px). These particles (106,905 particles) were finally refined and post-processed in RELION 4.0 with a mask on the N-terminal region. The resulting map was used for the refinement of an atomic model for the N-terminal region. The PDB 5EHD, which was used as a starting model, was docked in the map and refined in Coot 0.9.8.1 [54] and REFMAC5 [55].

Binding energy calculations

The peptide: protein binding energy was computed using MM/GBSA (Molecular Mechanics / Generalized Born Surface Area) method [56] as obtained from the following equations:

$$\Delta G_{Binding} = [G]_{complex} - [G]_{protein} - [G]_{peptide}$$

$$G = E_{bon} + E_{vdw} + E_{ele} + G_{pol} + G_{npol}$$

E_{bon} is composed of three bonded (bond, angle and dihedral) energy terms. E_{vdw} and E_{elec} are the van der waals and electrostatic non-bonded interaction components respectively. These energies are calculated using Molecular Mechanics (MM) force field expressions of AMBER. G_{pol} is the polar solvation energy which is obtained by solving the Generalized Born (GB) solvation model. G_{npol} is the non-polar solvation energy estimated through an empirical linear relationship (γ*SASA), where "γ" is the surface-tension and "SASA" is the Solvent Accessible Surface Area. The complex state structures were used to generate the corresponding free states of the protein and peptide. The continuum solvent environment was represented with an implicit GB model (IGB=2). The internal dielectric constant for the protein/peptide and the solvent was set to 1 and 80 respectively, $\gamma = 0.0072$ kcal/mol/$Å^2$ and the salt-concentration was set to 0.15 mM. The MMPBSA.py script [57] available through the AMBER18 suite of programs was used to carry out the calculations.

Light microscopy and fluorescence microscopy

Unlabeled NPM1 at 10 µM in 150 mM NaCl, 2 mM DTT, in 10 mM Tris pH 7.5 with or without basic peptides at concentrations of 10 or 100 µM were added to Corning 3651 polystyrene 96-well plates (Corning, USA). For light microscopy imaging, the mixtures were incubated at room temperature for 1 h and the liquid-liquid phase separation images in brightfield mode were recorded using a Zeiss Cell Observer microscopy instrument (Carl Zeiss AG, Germany) at a final magnification of 400x using LD Plan Neofluor 0.6 Corr Air objective. Images were recorded from initial focus point ±40 µm with a step of 1 µm.

For DroProbe imaging, 20 µM of 1,2-bis[4-(3-sulfonatopropoxyl) phenyl]-1,2-diphenylethene (DroProbe, AIEgen Biotech) was added to NPM1 in an 18 well chamber slide and incubated at room temperature for 5 min. Fluorescence microscopy images were acquired using a Nikon Eclipse Ti series inverted microscope (Nikon) equipped with Crest X-light V2 series confocal unit (Nikon), using 395 nm excitation wavelength and 3% laser power. Images were acquired using an S Plan ELWD 60X/0,70 oil immersion objective (Nikon) and a Zyla sCMOS camera (Andor). NIS-Elements Advanced Research 4.60.00 64-bit software (Nikon) was used for image analysis.

Tryptophan fluorescence spectroscopy

c-MYC *Pu24I* Quadruplex DNA (TGA GGG TGG IGA GGG TGG GGA AGG) was diluted in 10 mM Tris, 100 mM KCl pH 7.4 to a final concentration of 100 µM. The quadruplex DNA was formed by denaturation at +95°C for 15 min followed by overnight cooling to room temperature in a heating block. For tryptophan fluorescence spectroscopy, the samples were incubated for 2 h at +37°C on a Tecan Spark 20M multimode reader (Tecan Instruments, Switzerland) using Corning 3642 polystyrene 96-well plates (Corning, USA) and then excited at 280 nm (bandwidth 10 nm) with emission recorded at 350 nm

(bandwidth 10 nm). The gain was set automatically and data were analyzed with the Magellan software package (Tecan Instruments, Switzerland). $NPM1_{240}$, $NPM1_{240\text{-}294}$ and quadruplex DNA were tested at concentrations of 30 μM each. Results are expressed as average ± standard deviation of three replicates.

Supplementary References

44. Abelein, A. *et al.* High-yield Production of Amyloid-β Peptide Enabled by a Customized Spider Silk Domain. *Sci. Rep.* **10**, 235 (2020).
45. Pettersen, E. F. *et al.* UCSF ChimeraX: Structure visualization for researchers, educators, and developers. *Protein Sci.* **30**, 70–82 (2021).
46. Keller, R. W. *et al.* The nuclear poly(A) binding protein, PABP2, forms an oligomeric particle covering the length of the poly(A) tail. *J. Mol. Biol.* **297**, 569–583 (2000).
47. Tegunov, D. & Cramer, P. Real-time cryo-electron microscopy data preprocessing with Warp. *Nat. Methods* **16**, 1146–1152 (2019).
48. de la Rosa-Trevín, J. M. *et al.* Scipion: A software framework toward integration, reproducibility and validation in 3D electron microscopy. *J. Struct. Biol.* **195**, 93–99 (2016).
49. Wagner, T. *et al.* SPHIRE-crYOLO is a fast and accurate fully automated particle picker for cryo-EM. *Commun. Biol.* **2**, 218 (2019).
50. De la Rosa-Trevín, J. M. *et al.* Xmipp 3.0: An improved software suite for image processing in electron microscopy. *J. Struct. Biol.* **184**, 321–328 (2013).
51. Sorzano, C. O. S. *et al.* XMIPP: A new generation of an open-source image processing package for electron microscopy. *J. Struct. Biol.* **148**, 194–204 (2004).
52. Punjani, A., Rubinstein, J. L., Fleet, D. J. & Brubaker, M. A. CryoSPARC: Algorithms for rapid unsupervised cryo-EM structure determination. *Nat. Methods* **14**, 290–296 (2017).
53. Scheres, S. H. W. A bayesian view on cryo-EM structure determination. *J. Mol. Biol.* **415**, 406–418 (2012).
54. Emsley, P., Lohkamp, B., Scott, W. G. & Cowtan, K. Features and development of Coot. *Acta Crystallogr. Sect. D Biol. Crystallogr.* **66**, 486–501 (2010).
55. Murshudov, G. N. *et al.* REFMAC5 for the refinement of macromolecular crystal structures. *Acta Crystallogr. Sect. D Biol. Crystallogr.* **67**, 355–367 (2011).
56. Genheden, S. & Ryde, U. The MM/PBSA and MM/GBSA methods to estimate ligand-binding affinities. *Expert Opin. Drug Discov.* **10**, 449–461 (2015).
57. Miller, B. R. *et al.* MMPBSA.py: An efficient program for end-state free energy calculations. *J. Chem. Theory Comput.* **8**, 3314–3321 (2012).
58. Chen, V. B. *et al.* MolProbity: All-atom structure validation for macromolecular crystallography. *Acta Crystallogr. Sect. D Biol. Crystallogr.* **66**, 12–21 (2010).
59. Liebschner, D. *et al.* Macromolecular structure determination using X-rays, neutrons and electrons: Recent developments in Phenix. *Acta Crystallogr. Sect. D Struct. Biol.* **75**, 861–877 (2019).

Table S1. Model and data statistics
The data was generated with MolProbity [58] in Phenix 1.20.1 [59]

Model		
Composition (#)		
Chains	5	
Atoms	3673 (Hydrogens: 0)	
Residues	Protein: 481 Nucleotide: 0	
Water	0	
Ligands	0	
Bonds (RMSD)		
Length (Å) (# > 4sigma)	0.007 (0)	
Angles (°) (# > 4sigma)	1.193 (0)	
MolProbity score	1.84	
Clash score	11.75	
Ramachandran plot (%)		
Outliers	0.22	
Allowed	1.56	
Favored	98.22	
Rama-Z (Ramachandran plot Z-score, RMSD)		
whole (N = 449)	0.39 (0.36)	
helix (N = 0)	--- (---)	
sheet (N = 198)	1.48 (0.35)	
loop (N = 251)	-0.82 (0.33)	
Rotamer outliers (%)	2.18	
Cbeta outliers (%)	0	
Peptide plane (%)		
Cis proline/general	37.5/0.0	
Twisted proline/general	0.0/0.0	
CaBLAM outliers (%)	2.64	
ADP (B-factors)		
Iso/Aniso (#)	3673/0	
min/max/mean		
Protein	42.43/302.83/130.98	
Nucleotide	---	
Ligand	---	
Water	---	
Occupancy		
Mean	1	
occ = 1 (%)	100	
0 < occ < 1 (%)	0	
occ > 1 (%)	0	
Data		
Box		
Lengths (Å)	71.71, 69.69, 60.09	
Angles (°)	90.00, 90.00, 90.00	
Supplied Resolution (Å)	2.5	
Resolution Estimates (Å)	Masked	Unmasked

d FSC (half maps; 0.143)	3.5	3.6
d 99 (full/half1/half2)	2.1/1.0/1.0	1.3/1.0/1.0
d model	3.5	3.6
d FSC model (0/0.143/0.5)	2.2/2.8/3.7	2.2/3.1/3.8
Map min/max/mean	-0.13268E-02/0.42573E-02/0.63599E-05	
Model vs. Data		
CC (mask)	0.66	
CC (box)	0.8	
CC (peaks)	0.56	
CC (volume)	0.64	
Mean CC for ligands	---	

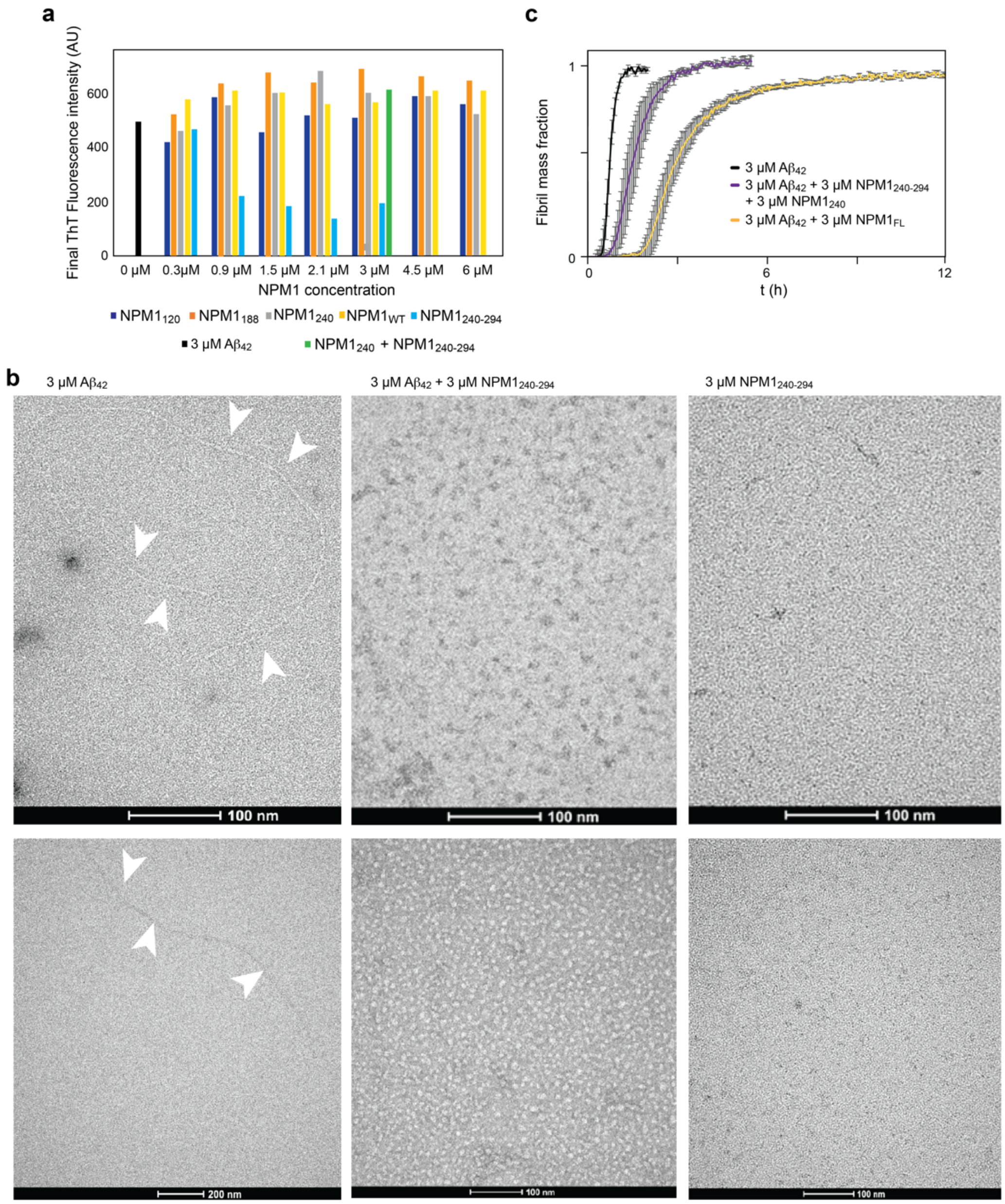

Figure S1. Effect of $NPM_{240-294}$ on $A\beta_{42}$ aggregation. (a) Final ThT fluorescence intensities of $A\beta_{42}$ for all NPM1 constructs at end-point (12 h) shown in Figure 1 indicate similar amounts of fibrils formed, except for incubation with $NPM_{240-294}$. (b) Negative stain electron microscopy of the reaction endpoints of $A\beta_{42}$ alone (left), $A\beta_{42}$ in the presence of $NPM_{240-294}$ (middle) and $NPM_{240-294}$ alone (right). Fibrils are observed only for $A\beta_{42}$ alone (white arrows). For $A\beta_{42}$ and $NPM_{240-294}$, some amorphous aggregates can be detected. Scale bars are shown at the bottom of each image. (c) ThT fluorescence curves for aggregation of $A\beta_{42}$ in the presence of $NPM1_{240}$ and $NPM1_{240-294}$ show that the addition of the NTD reduces chaperone activity of the CTD, also compared to FL NPM1. (b)

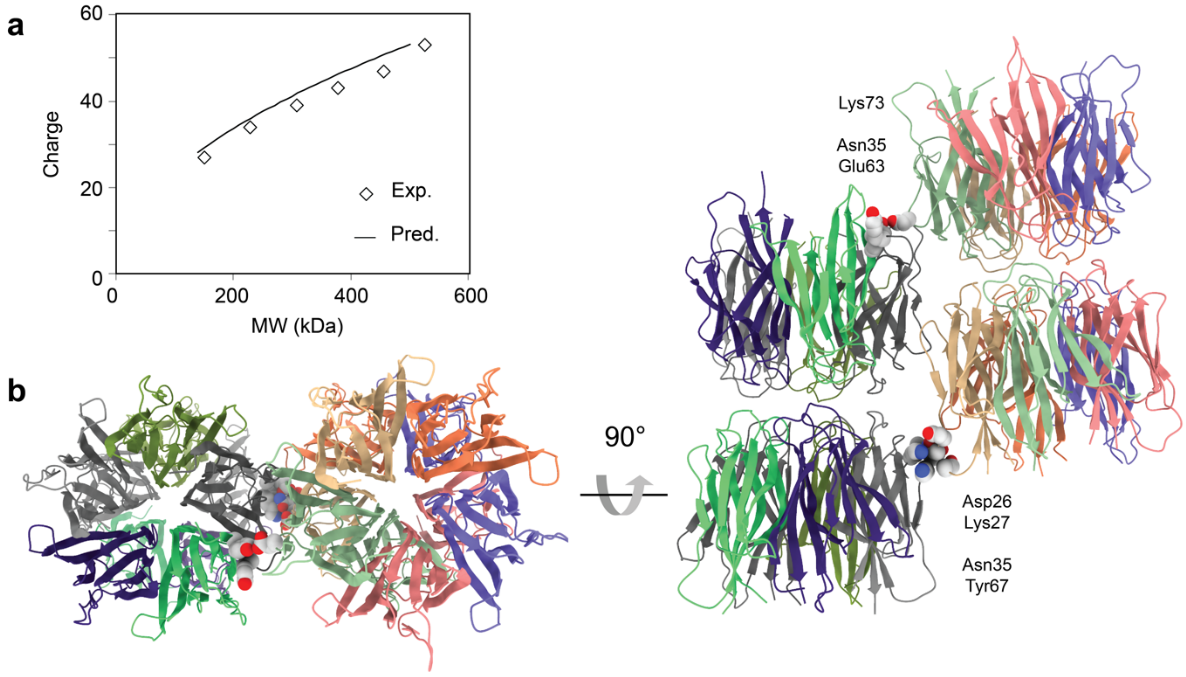

Figure S2. Ordered oligomerization of NPM_{120}. (a) Plotting the average charge of NPM_{120} oligomers as a function of molecular weight reveals a good correlation with the charge expected for globular protein (solid line). (b) Crystal packing of NPM1 NTDs shows end-to-end pentamers making salt bridges with neighboring oligomers (PDB ID 5EHM). Similar contacts could mediate multimerization of NPM_{120} observed by MS. Note stabilization of the acidic A1 tract (residues 34-39) through crystal contacts.

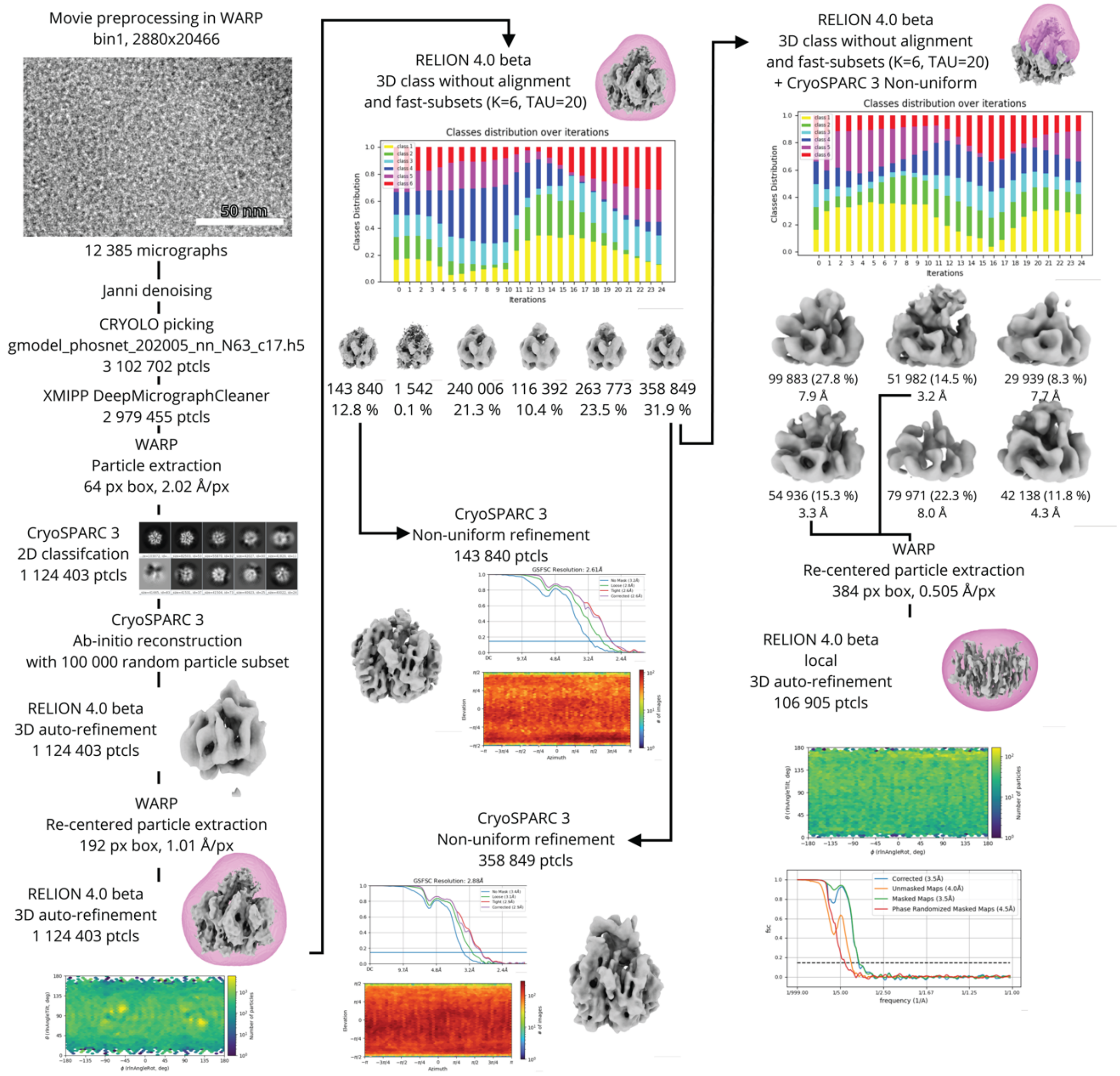

Figure S3. Cryo-EM analysis strategy for FL NPM1. The data analysis and density reconstruction strategy for NPM1.

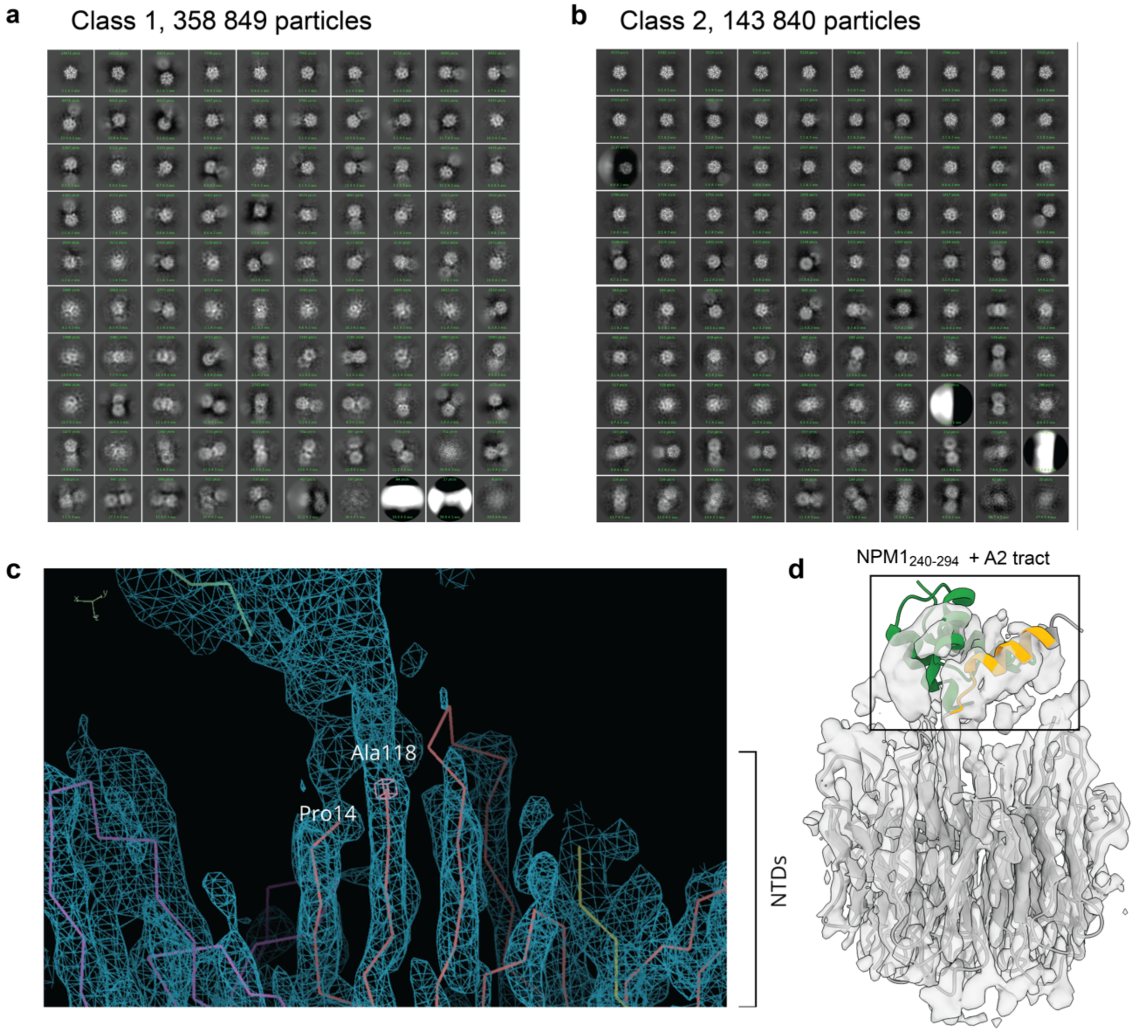

Figure S4. Cryo-EM analysis of NPM1 particles. (a) Particles in class 1 nearly always include one or two nearby particles that correspond to other NTD pentamers. (b) Particles in class 2 rarely have neighboring particles. (c) Fitting of the class 1 density map shows that the extra density above the NTD is connected to one protomer via its A2 tract, starting at residue 119. (d) Placement of the top-scoring CTD-A2 complex shows reasonable agreement between the location of the A2 tract and the CTD with the additional density in class 1 particles. The A2 tract, connected to the NTD, is shown in orange, the CTD in green. However, the A2-CTD complex can be fitted in multiple positions.

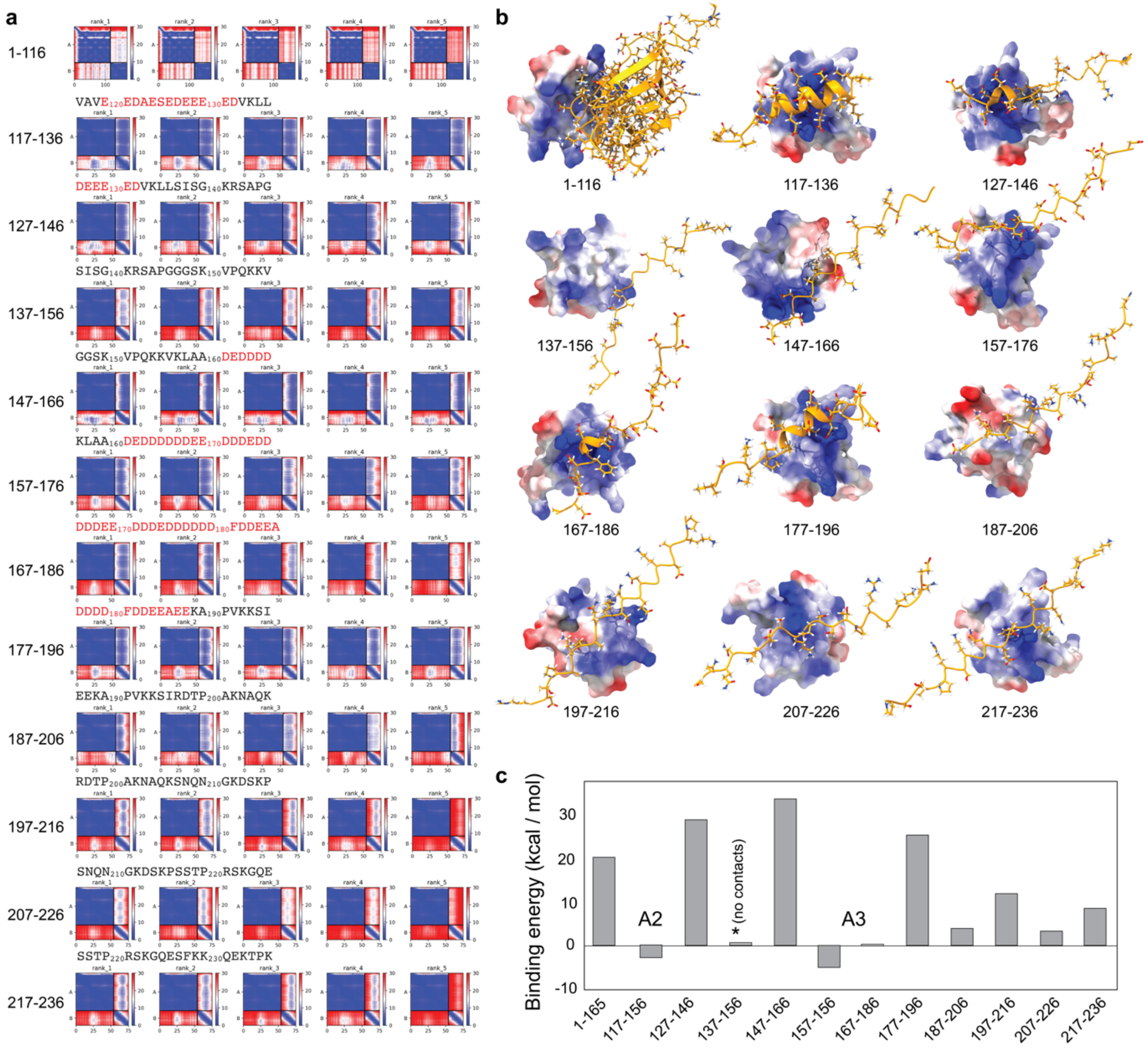

Figure S5. AF docking of the NTD and overlapping peptides covering the IDR to the CTD. (a) PAE plots show well-defined complexes between the CTD and peptides covering the A2 or A3 tracts. (b) The top-scoring models for each CTD-peptide complex are shown as electrostatic surfaces (CTD) and organe ribbons (peptides). Note that residues 136-154 make no contacts with the CTD. (c) Free energy calculations show weakly favorable interactions between the A2 and A3 tracts and the CTD.

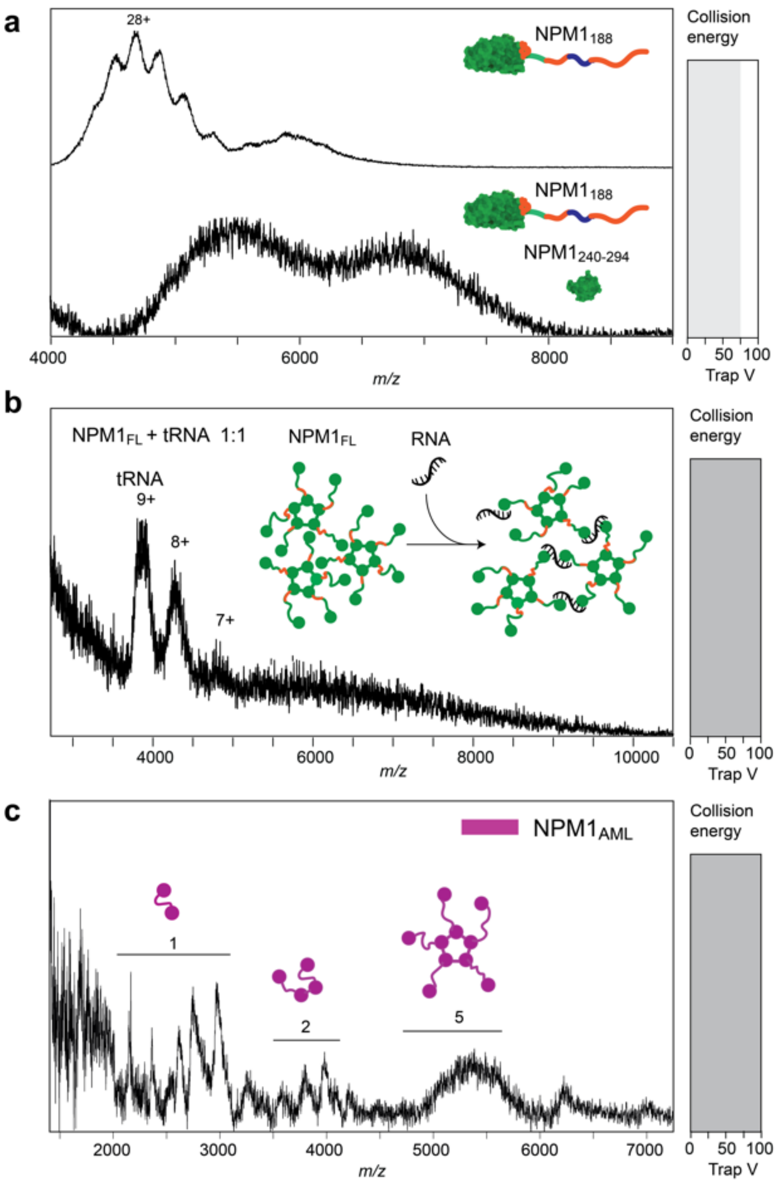

Figure S6. Interactions of wt and mutant NPM1 studied by MS. (a) Native MS of $NPM1_{188}$ in the absence (top) or the presence (bottom) of $NPM1_{240\text{-}294}$ shows a shift to the higher *m/z* region as well as significant peak broadening. (b) Addition of an equimolar amount of tRNA to NPM1 results in complete absence of NPM1 signal in mass spectra, consistent with cross-linking of NPM1 by tRNAs into stable oligomers. (c) Native MS and AF predictions of NPM1 lacking residues 284-294 in the CTD. Native mass spectra at a collision voltage of 100 V show Pentamers and smaller oligomers, comparable to FL wild-type NPM1. The AF models of the CTD of the AML variant (pink) show only minor changes in binding to the A1 and A2 tracts compared to the wild-type CTD (green).

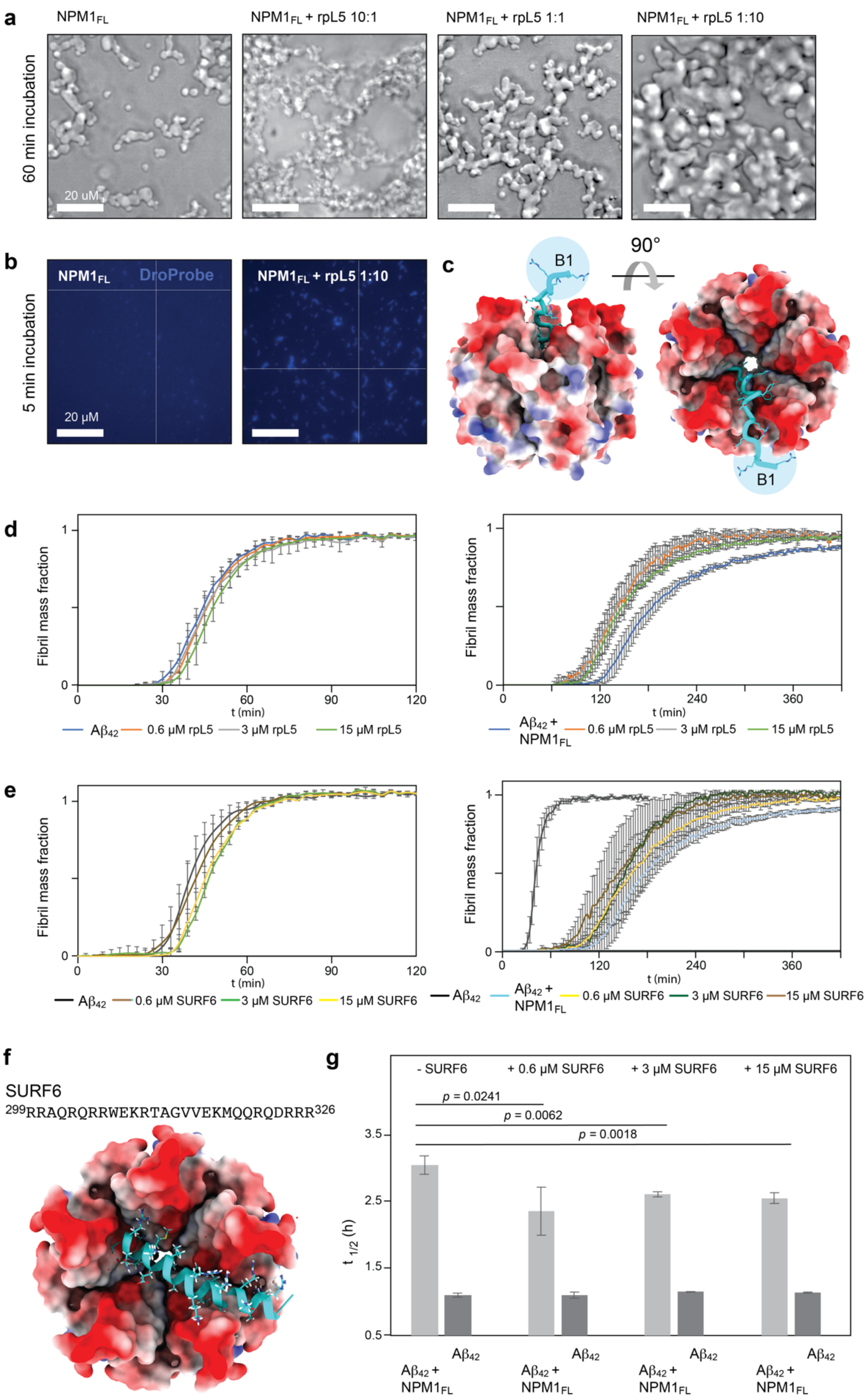
a
NPM1FL
NPM1FL + rpL5 10:1
NPM1FL + rpL5 1:1
NPM1FL + rpL5 1:10
60 min incubation
20 uM
b
5 min incubation
NPM1FL
DroProbe
NPM1FL + rpL5 1:10
20 μM
c
B1
90°
B1
d
Fibril mass fraction
t (min)
Aβ42
0.6 μM rpL5
3 μM rpL5
15 μM rpL5
Aβ42 + NPM1FL
e
Fibril mass fraction
t (min)
Aβ42
0.6 μM SURF6
3 μM SURF6
15 μM SURF6
Aβ42 + NPM1FL
f
SURF6
299RRAQRQRRWEKRTAGVVEKMQQRQDRRR326
g
- SURF6
+ 0.6 μM SURF6
+ 3 μM SURF6
+ 15 μM SURF6
p = 0.0241
p = 0.0062
p = 0.0018
t 1/2 (h)
Aβ42
Aβ42 + NPM1FL

Figure S7. AF predictions and Aβ_{42} aggregation assays of NPM1 bound to rpL5$_{22\text{-}37}$ and SURF6$_{299\text{-}326}$. (a) Light microscopy of FL NPM1 after incubation for 60 min alone or in the presence of rpL5$_{22\text{-}37}$ at ratios of 10:1, 1:1, or 1:10 shows an increase in number and size of NPM1 assemblies. (b) DroProbe fluorescence microscopy images of freshly dissolved NPM1 show an increase in viscous assemblies (blue) in presence of a ten-fold excess of rpL5$_{22\text{-}37}$. (c) Left: ThT fluorescence curves for Aβ_{42} alone as well as in the presence of rpL5$_{22\text{-}37}$ show no significant effect on fibril formation. Right: ThT fluorescence curves for Aβ_{42} in the presence of FL NPM1 show reduced chaperone activity in the presence of rpL5$_{22\text{-}37}$. (d) Left: ThT fluorescence curves for Aβ_{42} alone as well as in the presence of SURF6$_{299\text{-}326}$ show no pronounced effect on fibril formation. Right: ThT fluorescence curves for Aβ_{42} in the presence of FL NPM1 indicate reduced chaperone activity in the presence of SURF6$_{299\text{-}326}$. (e) AF prediction for the complex between NTD and SURF6$_{299\text{-}326}$ indicate binding of the SURF6 peptide to the acidic side of the NPM1 pentamer. The NTD pentamer is rendered as electrostatic surface, the SURF6 peptide is shown as ribbon in turquoise with basic residues as sticks. (f) $T_{1/2}$ of Aβ_{42} aggregation curves reveal that SURF6$_{299\text{-}326}$ has no pronounced effect on Aβ_{42} aggregation alone, but significantly reduces the chaperone-like activity of FL NPM1. Error bars indicate the standard deviation of n=4 repeats. Significance was calculated using Student's T-Test for paired samples with equal variance.

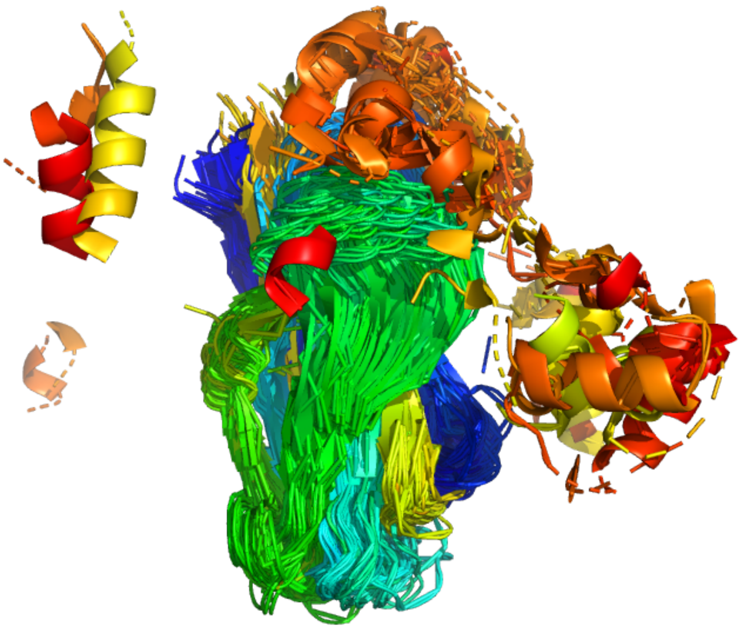

Figure S8. AF models for 29 homologs of NPM1 with identical domain architectures. Models are colored from N- to C-terminus and only non-disordered residues of the CTDs that are within a 4Å distance cut-off from the NTD are shown.
